# The Evaluation of Dipeptidyl Peptidase (DPP)-IV, α-Glucosidase and Angiotensin Converting Enzyme (ACE) Inhibitory Activities of Whey Proteins Hydrolyzed with Serine Protease Isolated from Asian Pumpkin (*Cucurbita ficifolia*)

**DOI:** 10.1007/s10989-014-9413-0

**Published:** 2014-06-01

**Authors:** Babij Konrad, Dąbrowska Anna, Szołtysik Marek, Pokora Marta, Zambrowicz Aleksandra, Chrzanowska Józefa

**Affiliations:** Department of Animal Products Technology and Quality Management, Wrocław University of Enviromental and Life Sciences, ul. Chełmońskiego 37/41, 51-630 Wrocław, Poland

**Keywords:** Asian pumpkin, Serine protease, Bioactive peptides, DPP-IV inhibitors, ACE inhibitors, α-Glucosidase inhibitors

## Abstract

In the present study, whey protein concentrate (WPC-80) and β-lactoglobulin were hydrolyzed with a noncommercial serine protease isolated from Asian pumpkin (*Cucurbita ficifolia*). Hydrolysates were further fractionated by ultrafiltration using membranes with cut-offs equal 3 and 10 kDa. Peptide fractions of molecular weight lower than 3 and 3–10 kDa were further subjected to the RP-HPLC. Separated preparations were investigated for their potential as the natural inhibitors of dipeptidyl peptidase (DPP-IV), α-glucosidase and angiotensin converting enzyme (ACE). WPC-80 hydrolysate showed higher inhibitory activities against the three tested enzymes than β-lactoglobulin hydrolysate. Especially high biological activities were exhibited by peptide fractions of molecular weight lower than 3 kDa, with ACE IC50 <0.64 mg/mL and DPP-IV IC50 <0.55 mg/mL. This study suggests that peptides generated from whey proteins may support postprandial glycemia regulation and blood pressure maintenance, and could be used as functional food ingredients in the diet of patients with type 2 diabetes.

## Introduction

Diabetes mellitus is recognized as a major health problem affecting millions of people (Go et al. 2013). This endocrine disorder, characterized by hyperglycemia, is associated with disturbances of carbohydrate, fat and protein metabolism resulting from altered insulin sensitivity and impaired insulin secretion (Schmidt and Hickey 2009). The contemporary therapeutic approach to diabetes is to decrease postprandial hyperglycemia, as strict glycemic control reduces the microvascular complications (Holman et al. 2008).

A recently introduced family of anti-diabetic drugs is based on the inhibition of dipeptidyl peptidase-IV (DPP-IV) and α-glucosidase (Deacon 2011). The former, DPP-IV (E.C. 3.4.14.5), has post-proline dipeptidyl aminopeptidase activity, with the specificity for removing X-Pro or X-Ala dipeptides from the N-terminus of polypeptides and proteins (Blanco et al. 1998; Hildebrandt et al. 2000). The endogenous physiological substrates of DPP-IV, two incretin hormones, glucagon-like peptide 1 (GLP-1) and glucose-dependent insulinotropic polypeptide (GIP) enhance glucose-induced insulin secretion during a meal. Actually, most of the secreted insulin is a result of the incretin response, mainly the combined action of GIP and GLP-1. However, both GIP and GLP-1, have a short half-life of 1–2 min, resulting from the cleaving activity of the enzyme (Psallas and Manes 2012). Therefore, the inhibition of the DPP-IV activity is seen as a promising treatment method in type 2 diabetes (Deacon 2011).

Another therapeutic approach in the management of postprandial hyperglycemia is based on the inhibition of α-glucosidase. The membrane-bound α-glucosidase (EC 3.2.1.20), present in the epithelial mucosa of the small intestine, cleaves glycosidic bonds in complex carbohydrate to release absorbable monosaccharides. The inhibition of α-glucosidase disables the release of free glucose from complex carbohydrates, thus decreasing postprandial blood glucose levels (Jaiswal et al. 2012).

Insulin resistance is often accompanied by hypertension (Psallas and Manes 2012; Buttar et al. 2005) which, together with a high plasma glucose level, is responsible for the long-term complications of diabetes, such as microvascular defects. As hypertension occurs when the angiotensin converting enzyme (ACE, EC 3.4.15.1) catalyzes the conversion of angiotensin I into angiotensin II. ACE inhibitors are commonly prescribed to diabetic patients, also to decrease the risk of other complications, including cancer, peptic ulcer and diabetic retinopathy (Skeggs et al. 1956; Ramos-Nino et al. 2008). However, although ACE inhibitors such as captopril, enalapril and lisinopril are commonly used in the treatment of patients with hypertension, heart failure or diabetic nephropathies, they have significant undesirable side effects and safe alternatives are needed (Song and White 2002; Ionescu et al. 2002).

Various reports suggest that dietary proteins could be natural precursors of the inhibitors of dipeptidyl peptidase (DPP)-IV, α-glucosidase and ACE (Huang et al. 2012; Lacroix and Li-Chan 2013, Silveira et al. 2013; Yu et al. 2011; Zambrowicz et al. 2013). Specific fragments of bioactive peptides have a beneficial impact on body functions, most notably on the nervous, immune, cardiovascular and digestive systems (Kitts and Weiler 2003; Haque and Chand 2008). The bioactive sequence in peptides may vary from 2 to 20 amino acid residues, and many peptides are known to have multi-functional properties (Haque and Chand 2008).

Dietary proteins may be an excellent source of biologically active peptides. These peptides, inactive within the sequence of the native protein, may be released both during digestion in the gastrointestinal tract and during food processing (Korhonen et al. 1998; Korhonen and Pihlanto-Leppälä 2006). Food protein hydrolysates are natural ingredients, and therefore they are believed to be safe for consumers when they are served as functional foods. Studies indicate the potential of using egg white protein hydrolysates as functional products with the anti-diabetic activity as the inhibitor of α-glucosidase (Yu et al. 2011). Peptides derived from sardine muscle hydrolysate, digested with *Bacillus licheniformis* alkaline protease expressed similar activity (Matsui et al. 1999). The α-glucosidase inhibitory activity of dairy protein hydrolysates has been reported recently (Lacroix and Li-Chan 2013).

Many milk-derived peptides reveal multifunctional properties, i.e. specific peptide sequences with two or more different biological activities. As shown by many researchers, both casein and whey protein may be an important source of peptides with bioactive properties. For example, whey proteins can be a source of dipeptidyl dipeptidase IV inhibitors (Tulipano et al. 2011). Milk protein-derived peptides are claimed to be health enhancing components that can be used to reduce the risk of disease or to enhance a certain physiological function.

Among the different classes of bioactive peptides, the antihypertensive peptides are the best known. ACE inhibitory peptides have been discovered in various food sources such as milk, gelatine, maize and soybean (Meisel 1997; Oshima et al. 1979; Miyoshi et al. 1991, Okamoto et al. 1995). Antihypertensive peptides have been found in processed dairy products. ACE inhibitors derived from milk proteins represent different fragments of casein (casokinins) or whey proteins (lactokinins) (Nakamura et al. 1995; Korhonen and Pihlanto-Leppälä 2006). Two potent ACE-inhibitory peptides from β-casein, f84–f86, which corresponds to Val–Pro–Pro, and f74–f76, which corresponds to Ile–Pro–Pro, and one from k-casein, f108–f110, which corresponds to Ile–Pro–Pro, were purified from the Japanese soft drink “Calpis”, made from bovine skim milk fermented with *Lactobacillus helveticus* and *Saccharomyces cerevisiae* (Nakamura et al. 1995). The results of Pihlanto’s research demonstrate the existence of several biologically active whey-derived peptides and hydrolysates (Pihlanto 2000).

Whey proteins are significantly resistant to hydrolysis and the use of enzymes significantly increases the cost of their production. One of the promising alternatives is the use of plant serine protease isolated from *C. ficifolia,* exhibiting attractive proteolytic properties towards casein, protein from corn gluten meal (CGM) or ovoalbumin (Illanes et al. 1985; Curotto et al. 1989; Pokora et al. 2014). The protease exhibits a very high and broad pH optimum with a maximum at 10.7 and is able to cleave four bonds in an endogenous serine proteinase inhibitor. The optimum temperature is 35 °C and optimum pH is 8.6 (Dryjański et al. 1990). Taking this into account we used serine protease from *C. ficifolia* to hydrolyze whey proteins to generate peptides with antidiabetic and antyhipertensive activities.

The aim of this study is to investigate peptides generated from whey proteins hydrolyzed by the non-commercial proteolytic enzyme obtained from Asian pumpkin as the natural sources of DPP-IV, α-glucosidase and ACE inhibitors that can be used as functional food ingredients for the complex management of type 2 diabetes and hypertension.

## Materials and Methods

### Isolation of the Enzyme

Serine protease was isolated from Asian pumpkin according to the method of Dryjański et al. (1990). After separating the peel from the seeds, the pulp was homogenized and centrifuged at 5,000×*g*, 20 min. Clear supernatant was added to solid ammonium sulphate in order to achieve 30 % saturation. The final precipitate was collected by centrifugation at 5,000×*g*, 20 min. Desalting was conducted by dialysis in water. The specific activity of the enzyme preparation was 4,411 U/g.

### Substrates

β-lactoglobulin was provided by Sigma (L3908) and the whey protein concentrate (WPC-80), manufactured from sweet whey and spray-dried, was provided by Davisco Foods Iternational, Inc.

### Determination of Enzyme Activity

Proteolytic activity of the serine protease was determined with the use of 2 % casein as a substrate in 0.1 M Tris–HCl at pH 8.6. The substrate was incubated with the enzyme for 10 min at 37 °C. After this time the reaction was stopped by the addition of 5 % trichloroacetic acid (TCA). The sample was then centrifuged and absorbance measured at λ = 280 nm. One unit of enzyme activity was defined as the amount of enzyme giving an increase in absorbance of 0.1 under conditions described above (Chrzanowska and Kołaczkowska 1998).

### Determination of Protein Content

Protein content was determined by colorimetric method of Lowry et al. (1951), using BSA (Sigma, P0834) as a standard.

### Hydrolysis of β-Lactoglobulin and WPC-80

Enzymatic hydrolysis of 1 % β-lactoglobulin and 1 % WPC-80 solution was conducted using serine protease isolated from Asian pumpkin at the dose of 150 U/mg of hydrolyzed protein. The reaction was carried out at 37 °C for 5 h in 0.1 M Tris–HCl buffer at pH 8.0. The hydrolysis was terminated by thermal inactivation (for biological activity determinations) or by the addition of 10 % trichloroacetic acid (TCA) (1:1 V/V).

### Ultrafiltration

5-hour hydrolysates were partially fractionated by ultrafiltration with Amicon Ultra-15 Millipore membranes with cut-offs equal 3 and 10 kDa. The separated permeates were vacuum concentrated.

### Reversed-Phase High Performance Liquid Chromatography (RP-HPLC)

Peptide profiles were determined by RP-HPLC with an Agilent 1100 Series system. The peptide preparations were solubilized in the even volume of phase A (0.1 % TFA in H_2_O) before loading on the chromatographic HPLC column (Zorbax Eclipse XDB-C18 Agilent column (50 × 4.6 mm). Separation was performed at a flow rate of 1 mL/min at 30 °C. Peptide fractions, varying in hydrophobicity, eluted from the column in linear gradient of phase B (0.1 % TFA in acetonitrile), were collected and lyophilized. Absorbance measurement was made at λ = 230 nm (DAD, G1315B). After resuspension of peptide fractions in Mili-Q water, their inhibitory activity was determined. Further peptide separation was performed using a Zorbax XDB-C18 Agilent column (250 × 4.5 mm). The absorbed peptides were eluted with a gradient (0–100 %) of phase B. Other analysis conditions are described above (Ardo and Gripon 1995).

### The Degree of Hydrolysis

The course of the hydrolysis was monitored by the determination of soluble peptide concentration in 5 % TCA in relation to the total protein. The concentration of the trichloroacetic acid-soluble product in the supernatant was measured spectrophotometrically at λ 280 nm (Spellman et al. 2003).

### The Free Amino Groups Content

The content of free amino groups (FAG) (μmol Gly/g) was determined using trinitrobenzene sulfonic acid (TNBS, Sigma) according to the method described by Kuchroo et al. (1983). Samples of hydrolysate were diluted with 0.1 mol/L borate buffer to the final volume of 2 mL, then mixed with 50 μL of TNBS reagent (0.03 mol/L) and incubated in the dark for 2 h at room temperature. The reaction was stopped by adding 2 mL of 0.1 mol/L sodium phosphate containing 1.5 mmol/L sodium sulfate and the absorbance was measured spectrophotometrically at λ 420 nm. The results were expressed as μmol Gly/g in relation to a standard curve prepared with the defined concentration of glycine.

### ACE-Inhibitory Activity

ACE-inhibitory activity (IC_50_) was assayed by the spectrophotometric method with the use of hippury-l-histydyl-l-leucine (HHL) (Sigma, H4884) as substrate (Miguel et al. 2007). HHL (5 mmol/L in 100 mmol/L potassium phosphate containing 300 mmol/L sodium chloride, pH 8.3), enzyme (ACE from rabbit lung, Sigma, A6778) and peptide solutions were incubated at 37 °C for 30 min. The reaction was stopped with the addition of 1 mol/L HCl. Conversion of HHL to hippurate and l-histidyl-l-leucine was quantified spectrophotometrically at λ 228 nm. The IC_50_ was defined as the concentration of inhibitor required to inhibit 50 % of the ACE activity.

### α-Glucosidase Inhibitory Activity

The α-glucosidase-inhibition assay was performed according to the method of Yu et al. (2011). α-Glucosidase from *Saccharomyces cerevisiae* (Sigma, G0660) hydrolyzed the substrate—*p*-nitro phenyl glucopyranoside (pNPG) (Sigma, N1377), and the thus produced *p*-nitro phenol can be measured through the absorbance at the λ 410 nm. The IC_50_ was defined as the concentration of inhibitor required to inhibit 50 % of the α-glucosidase activity under the assay conditions.

### DPP-IV-Inhibitory Activity

DPP-IV-inhibitory activity (IC_50_) was determined using a modified method of Lacroix and Li-Chan (2013). DPP-IV from porcine kidney was provided by Sigma (D7052). The lyophilized peptide fractions were resuspended in 0.1 M/L Tris–HCl buffer, pH 8.0. The test sample (25 μL) was preincubated with the equal volume of the substrate Gly-Pro-*p*-nitroanilide (1.6 mM) (Sigma, G0513) at 37 °C for 10 min. Afterwards, 50 μL of DPP-IV (0.01 U/mL, in 0.1 M/L Tris–HCl buffer, pH 8.0) was added and the mixture was incubated at 37 °C for 60 min. The reaction was stopped by the addition of 100 μL of 1 M/L sodium acetate buffer, pH 4.0. The released *p*-nitroanilide as a hydrolysis product was measured at λ 405 nm. The IC_50_ was defined as the concentration of inhibitor required to inhibit 50 % of the DPP-IV activity under the assay conditions.

### Statistical Analysis

All assays were conducted in triplicate. The results were analyzed using analysis of variance (ANOVA) in Statistica 7.0 software. Significance was set at the level of *p* ≤ 0.05.

## Results and Discussion

### Extent of Hydrolysis

**Fig. 1:**
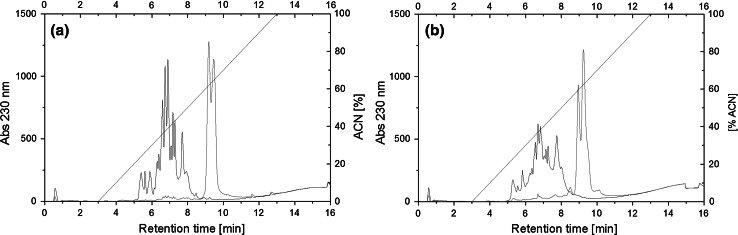
RP-HPLC profiles of peptide fractions (black) obtained after 5 h hydrolysis of **a** β-lactoglobulin and **b** WPC-80 with serine protease isolated from Asian pumpkin (*Cucurbita ficifolia*) introduced at the dose of 150 U/mg. Undigested 1 % protein solution was use as control of hydrolysis (*red*) (Color figure online)

The course of the enzymatic hydrolysis of the whey proteins was monitored by the determination of the degree of hydrolysis [DH] (%). The determined DH for β-lactoglobulin and WPC-80 after 5 h hydrolysis reached 27.3 % and 30.5 %, respectively. During the protein degradation in all hydrolysates also the proportional increase in the FAG content was observed. The final concentration of released FAG reached 2516 µmol Gly/g and 2924 µmol Gly/g for β-lactoglobulin hydrolysate and WPC-80 hydrolysate, respectively. The progress of hydrolysis was also confirmed by RP-HPLC peptide profiles analysis (Fig. 1). On the chromatograms of hydrolysates obtained after 5 h of reaction, the presence of subfractions was noted, which were eluted from the column at low concentration of acetonitrile and varied in terms of hydrophobicity. The majority of peptides were eluted at ACN concentration of 35–40 %.

**Fig. 2:**
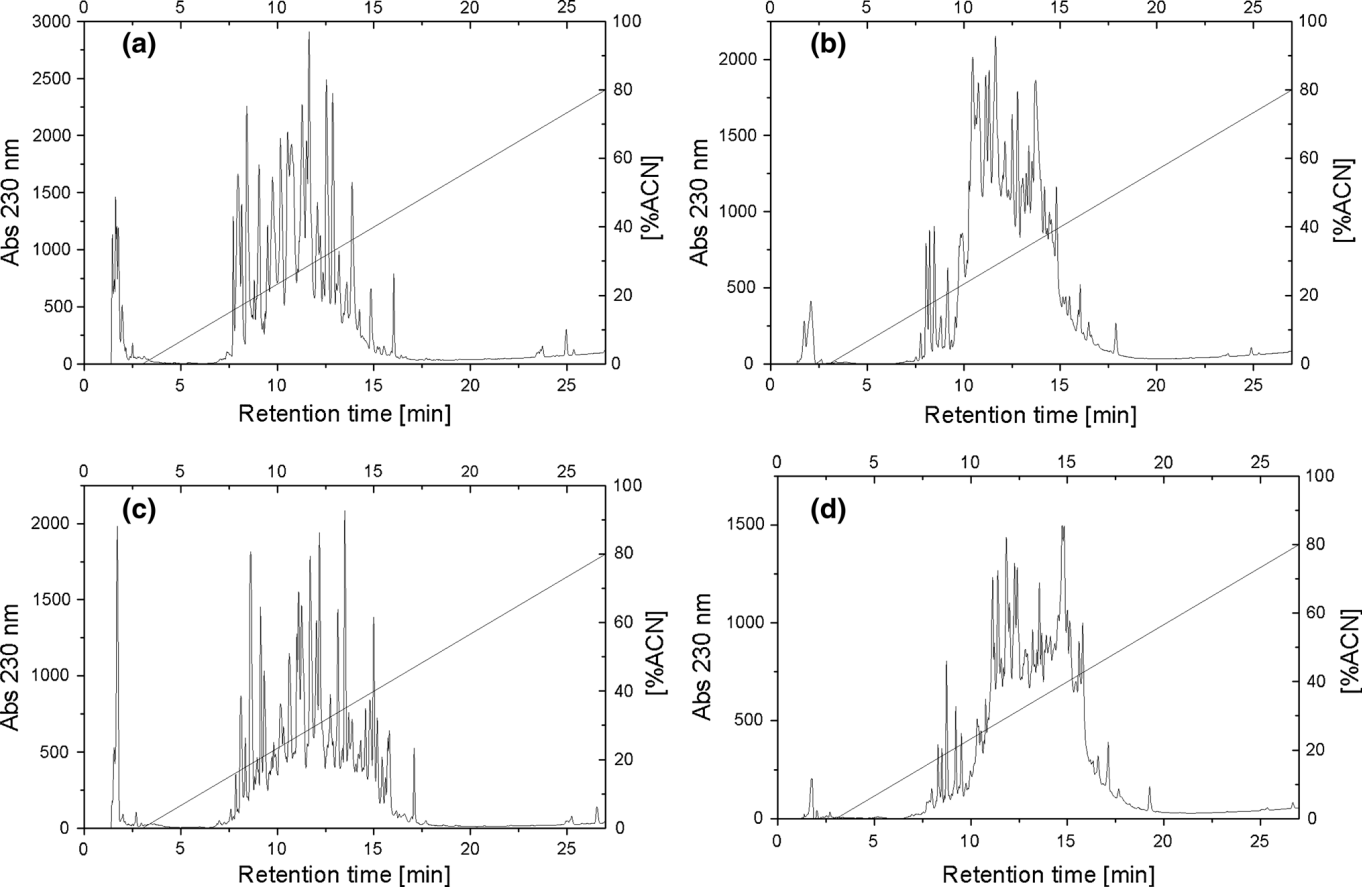
RP-HPLC peptide profiles of permeates obtained after ultrafiltration **a** β- lactoglobulin <3 kDa, **b** β-lactoglobulin 3–10 kDa, **c** WPC-80 <3 kDa, WPC-80 3–10 kDa

Hydrolysates of β-lactoglobulin and WPC-80 were ultrafiltrated with 3 and 10 kDa membranes, and thus permeates of the molecular mass below 3 kDa and ranging from 3 to 10 kDa were distinguished. Each permeate was further fractionated using the RP-HPLC method (Fig. 2), which resulted in 14 and 12 fractions for β-lactoglobulin and 15 and 20 for WPC-80 hydrolysates, respectively.

### DPP-IV Inhibition

**Fig. 3:**
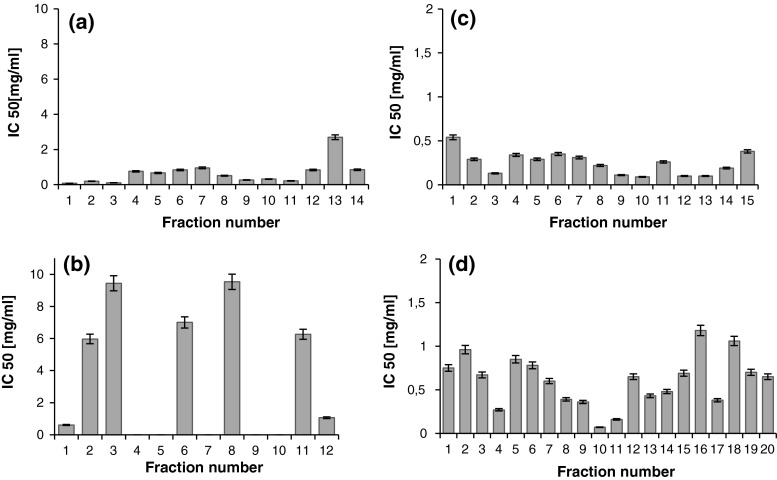
DPP-IV inhibitory activity of β-lactoglobulin (**a**, **b**) and WPC derived peptide fractions (**c**, **d**). β-lactoglobulin fractions of molecular mass <3 kDa (**a**), 3–10 kDa (**b**). WPC fractions of molecular mass <3 kDa (**c**), 3–10 kDa (**d**). DPP-IV inhibitory activity was reported as IC50 i.e. the concentration of the inhibitor required to inhibit 50 % of the DPP-IV activity under the assay conditions

The vast majority of all collected fractions revealed significant ability to inhibit the activity of DPP-IV (Fig. 3a–d). The only exception were five β-lactoglobulin-derived peptide fractions within the 3–10 kDa range (Fig. 3b). Although the inhibition of DPP-IV activity was observed in all the fractions obtained from the WPC-80 hydrolysate, the peptide fractions below 3 kDa showed the greatest potency at IC_50_ < 0.55 mg/mL (Fig. 3c). However, it was fraction no. 10 (WPC-80 peptides in the 3–10 kDa range) exhibited the highest activity against this enzyme (Fig. 3d). A concentration of 0.07 mg/mL was required in order to decrease the activity of the enzyme to 50 %. The results obtained for fractions derived from WPC with 3–10 kDa were different than the results obtained for β-lactoglobulin (Fig. 3b, d).

It is possible that peptides released from other whey protein components contributed to the activity detected for the hydrolysate obtained from WPC. In the research of Lacroix and Li-Chan (2013) all the products of the peptic treatment of whey protein isolate: β-lactoglobulin, α-lactalbumin, bovine serum albumin and lactoferrin present at a concentration of 500 µg/mL caused significant inhibition of the DPP-IV enzyme. The IC_50_ values defined in the cited research were: 0.075 and 1.28 mg/mL for WPI and β-lactoglobulin hydrolysates, respectively (Lacroix and Li-Chan, 2013).

Another study also confirmed that whey proteins have peptide sequences with potential DPP-IV inhibitory activity. Tryptic hydrolysate of β-lactoglobulin displayed the inhibitory activity that reached IC_50_ = 210 μM (Agyei and Danquah, 2011). This is in the line with results obtained by Silveira et al. (2013), who reported that peptide fractions isolated from tryptic hydrolysate of WPC can be an effective DPP-IV inhibitor. Among six peptide fractions which were previously separated by RP-HPLC the most potent fragment, IPAVF (IC_50_ = 44.7 μM), corresponded to β-lactoglobulin f(78–82) (Silveira et al. 2013).

### α-Glucosidase Inhibition

**Fig. 4:**
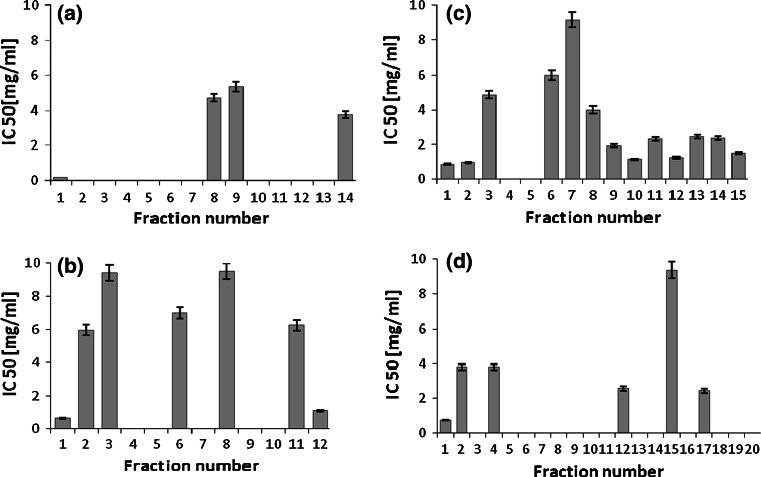
α-Glucosidase inhibitory activity of β-lactoglobulin (**a**, **b**) and WPC derived peptide fractions (**c**, **d**). β-lactoglobulin fractions of molecular mass <3 kDa (**a**), 3–10 kDa (**b**). WPC fractions of molecular mass <3 kDa (**c**), 3–10 kDa (**d**). α-Glucosidase inhibitory activity was reported as IC50 i.e. the concentration of the inhibitor required to inhibit 50 % of the DPP-IV activity under the assay conditions

The whey protein hydrolysates obtained by the whey protein degradation with the use of serine protease from *C. ficifolia* were also assessed for their inhibitory activity against α-glucosidase (Fig. 4a–d). Among fifteen peptide fractions derived from the WPC-80 hydrolysate with the molecular mass below 3 kDa, thirteen exhibited α-glucosidase inhibitory activity (Fig. 4c). Within this group six fractions showed the greatest potency with the IC_50_ values below 2.0 mg/mL. However, when we compared the results with those of β-lactoglobulin peptide fractions of the same molecular mass range, only four fractions displayed the inhibiting activity. The relatively low inhibitory activity was surprising because β-lactoglobulin is the major protein fraction in whey. In addition, in the paper of Lacroix and Li-Chan (2013), α-lactalbumin, lactoferrin and serum albumin hydrolysates obtained by peptic digestion were able to inhibit the activity of α-glucosidase.

The only study on α-glucosidase inhibitory activity of whey protein hydrolysates was conducted by Lacroix and Li-Chan (2013). The inhibitory activity towards α-glucosidase was observed only in case of WPI (IC_50_ = 4.5 mg/mL) and β-lactoglobulin (IC_50_ = 3.5 mg/mL). The different levels of this activity in their study might have resulted from the use of rat intestinal α-glucosidase in the assay (Lacroix and Li-Chan, 2013). Some synthetic inhibitors show different ability to inhibit the activity of α-glucosidase depending on the enzyme origin. They strongly affect the activity of mammalian α-glucosidase, but have little inhibitory effect on baker’s yeast α-glucosidase (Oki et al. 1999). On the other hand, some food products such as yogurt, chicken essence and fish sauce, exhibit inhibitory activity only against yeast α-glucosidase (Oki et al. 1999). Moreover, Lacroix and Li-Chan in their study used pepsin in the hydrolysis. Probably the different traits of pepsin and serine protease from *C. ficifolia* were the most important factor affecting the biological activity of hydrolysates.

Our results indicate that β-lactoglobulin-derived peptides of the molecular mass 3–10 kDa coincide with those obtained from WPC-80. However, the WPC-80 derived peptides showed greater potency than those originated from β-lactoglobulin. Five of the six fractions had IC_50_ below 4.0 mg/mL (Fig. 4d). These observations are in line with results obtained by Matsui et al. (1999) and by Yu et al. (2011) who reported the similar levels of α-glucosidase inhibitory activity of other food protein hydrolysates. Matsui et al. (1999) showed that an alkaline protease hydrolysate from sardine muscle was able to inhibit the carbohydrate-hydrolyzing enzyme with IC_50_ at 48.7 mg/mL. More recently, the hexapeptide Arg-Val-Pro-Ser-Leu-Met (IC_50_ = 23.07 μM or ~0.016 mg/mL) and the pentapeptide Thr-Pro-Ser-Pro-Arg (IC_50_ = 40.02 μM or ∼0.022 mg/mL), derived from egg white protein, were reported to possess α-glucosidase inhibitory activity.

### ACE Inhibition

**Fig. 5:**
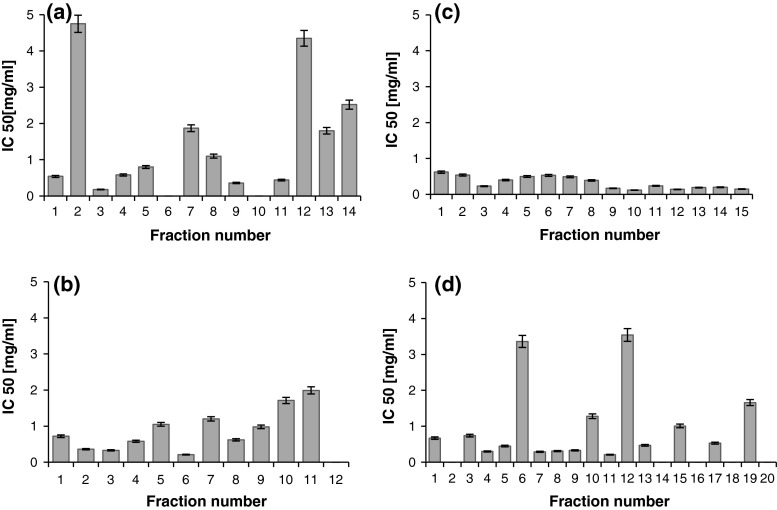
ACE inhibitory activity of β-lactoglobulin (**a**, **b**) and WPC derived peptide fractions (**c**, **d**). β-lactoglobulin fractions of molecular mass <3 kDa (**a**), 3–10 kDa (**b**). WPC fractions of molecular mass <3 kDa (**c**), 3–10 kDa (**d**). ACE inhibitory activity was reported as IC50 i.e. the concentration of the inhibitor required to inhibit 50 % of the DPP-IV activity under the assay conditions

The whey protein hydrolyzed by serine protease from *C. ficifolia* were also tested for their inhibitory activity against the ACE (Fig. 5a–d). Vast majority of all the collected fractions exhibited the ability to inhibit ACE. The highest activity was observed in case of the WPC-80 hydrolysate with the peptide fractions up to 3 kDa (Fig. 5c), showing the greatest potency at IC_50_ below 0.65 mg/mL. A relatively lower inhibitory activity of the hydrolyzed β-lactoglobulin samples was observed. Six fractions originated from β-lactoglobulin peptides of molecular masses below 3 kDa, with IC_50_ below 0.8 mg/mL (Fig. 5 a). The inhibitory activity of six fractions ranged between 1.1 and 4.75 mg/mL, whilst two fractions did not inhibit ACE. In comparison, WPC-80 derived peptides of molecular size 3–10 kDa contained five inactive fractions (Fig. 5d). However, the determined activity expressed by IC_50_ values in ten preparations did not exceed the level of 0.8 mg/mL. In case of β-lactoglobulin derived peptides of molecular size 3–10 kDa only one preparation did not exhibit inhibitory activity against the ACE. Six fractions were able to decrease the activity of the enzyme to 50 % of the maximum at a concentration below 0.8 mg/mL (Fig. 5b).

According to Hartmann and Meisel (2007), ACE-inhibitory peptides are generally short-chain and contain polar amino acid residues in their structure. Other authors demonstrate that ACE inhibition is mainly attributable to peptides with molecular masses lower than 3 kDa (Miguel et al. 2004; Saiga et al. 2006). The hypotensive peptides Val-Pro-Pro and Ile-Pro-Pro, for example, can be released from β-casein and κ-casein by enzymes obtained from *Lactobacillus helveticus* (Miguel et al. 2004). Other authors report that ACE-inhibitory activity is stronger in case of dipeptides, such as -His-Leu, -Phe-Arg, or -Ala-Pro (Saiga et al. 2006; Miguel et al. 2007). On the other hand longer-chain peptides present in egg-white peptic hydrolysate (RADHPFL and YAEERYPIL) can also exert ACE-inhibitory activity. Saiga et al. (2006) also found an ACE-inhibitory long-chain peptide (Gly-Phe-Hyp-Gly-Thr-Hyp-Gly-Leu-Hyp-Gly-Phe), in an extract of chicken breast muscle hydrolyzed by gastric enzymes (trypsin-chymotrypsin and small intestinal enzymes).

Most of the tested peptides with inhibitory properties against DPP-IV were able to inhibit ACE simultaneously. This was exhibited by each of the fifteen WPC-80 derived peptide fractions up to 3 kDa. Moreover, within this group thirteen preparations were capable of inhibiting the activity of the three tested enzymes. The triple inhibition was observed also in the case of four peptide fractions of β-lactoglobulin up to 3 kDa, four β-lactoglobulin fractions from 3 to 10 kDa and five WPC-80 fractions from 3 to 10 kDa.

## Conclusion

The results of the present study demonstrate that hydrolysis of either β-lactoglobulin or WPC-80 by the noncommercial serine protease isolated from Asian pumpkin (*Cucurbita ficifolia*) resulted in the production of peptides with inhibitory properties against DPP-IV, α-glucosidase and ACE. These findings suggest that whey protein hydrolysates with such inhibitory traits may potentially improve blood glucose regulation by means of their ability to slow both the inactivation of the incretin hormones and the intestinal digestion of carbohydrates and also decrease other complications associated with diabetes. However, further research is needed for the better understanding of molecular mechanisms of action of the defined peptides. In addition, clinical studies are necessary in order to confirm the efficiency and bioavailability of whey protein-derived peptides in humans.
